# Association of pulmonary transit time by cardiac magnetic resonance with heart failure hospitalization in a large prospective cohort with diverse cardiac conditions

**DOI:** 10.1186/s12968-023-00963-8

**Published:** 2023-10-12

**Authors:** J. Jane Cao, Niloofar Fouladi Nashta, Jonathan Weber, Ruqiyya Bano, Michael Passick, Y. Joshua Cheng, William Schapiro, Marie Grgas, Kathleen Gliganic

**Affiliations:** 1 Division of Cardiac Imaging, St. Francis Hospital & Heart Center, 100 Port Washington Blvd., Roslyn, NY 11576 USA; 2 DeMatteis Cardiovascular Institute, St. Francis Hospital & Heart Center, Roslyn, NY USA; 3 Sol Price School of Public Policy and Leonard D. Schaeffer Center for Health Policy and Economics, University of Southern California, Los Angeles, CA USA

**Keywords:** Pulmonary transit time, Cardiac MRI, Heart failure, Left ventricular ejection fraction

## Abstract

**Background:**

Longer pulmonary transit time (PTT) is closely associated with hemodynamic abnormalities. However, the implications on heart failure (HF) risk have not been investigated broadly in patients with diverse cardiac conditions. In this study we examined the long-term risk of HF hospitalization associated with longer PTT in a large prospective cohort with a broad spectrum of cardiac conditions.

**Methods:**

All subjects were prospectively recruited to undergo cardiac magnetic resonance (CMR). The dynamic images of first-pass perfusion were acquired to assess peak-to-peak pulmonary transit time (PTT) which was subsequently normalized to RR interval duration. The risk of HF was examined using Cox proportional hazards models adjusted for baseline confounding risk factors.

**Results:**

Among 506 consecutively consented patients undergoing clinical cardiac MR with diverse cardiac conditions, the mean age was 63 ± 14 years and 373 (73%) were male. After a mean follow up duration of 4.5 ± 3.0 years, 70 (14%) patients developed hospitalized HF and of these 6 died. A normalized PTT ≥ 8.2 was associated with a significantly increased adjusted HF hazard ratio of 3.69 (95% CI 2.02, 6.73). The HF hazard ratio was 1.26 (95% CI 1.18, 1.33) for each 1 unit increase in PTT which was higher among those preserved (1.70, 95% CI 1.20, 2.41) compared to those with reduced left ventricular ejection fraction (< 50%) (1.18, 95% CI 1.09, 1.27). PTT remained a significant risk factor of hospitalized HF after additional adjustment for N-terminal pro-hormone brain natriuretic peptide (NT-proBNP) or left ventricular global longitudinal strain with additionally demonstrated incremental model improvement through likelihood ratio testing.

**Conclusions:**

Our findings support the role of PTT in assessing HF risk among patients with broad spectrum of cardiac conditions with reduced as well as preserved ejection fraction. Longer PTT duration is an incremental risk factor for HF when baseline global longitudinal strain and NT-proBNP are taken into consideration.

## Introduction

There is an extensive body of work examining the central circulation time spanning nearly a century using a predominantly invasive approach in the classic literature [1–7] and non-invasive modalities in the modern era [8–17]. Most investigations have focused on the transit time between the right and left circulation, commonly referred as pulmonary transit time (PTT) where a longer duration has been associated with reduced cardiac function or stroke volume, as well as pulmonary hypertension and heart failure [8–17]. To date, the prognostic value of PTT has begun to emerge in selected cardiac conditions [18–22] in determining adverse clinical outcomes. As a biomarker, PTT is relatively easy to acquire. However, longer PTT duration is not specific to a particular cardiac disease as many conditions associated with altered cardiopulmonary hemodynamics can influence the duration of PTT including ventricular preload, blood volumes, global ventricular function, valvular function, and pulmonary diseases that affect the pulmonary circulation [1–19]. Since all these factors may also contribute to the development of heart failure, we examined the long-term risk of heart failure hospitalization associated with longer PTT duration in a large prospective patient cohort with broad spectrum of cardiac conditions.

## Methods

### Participant recruitment

The study protocol was approved by the St. Francis Hospital Institutional Review Board. Participants were recruited prospectively and written informed consent was obtained from all. Most of the participants were recruited from those who were referred for clinical cardiovascular magnetic resonance (CMR). The exclusions were the same for all participants, including impaired renal function with glomerular filtration rate (GFR) less than 45 ml/min/1.73 m^2^; claustrophobia; pacemaker/defibrillator implantation or other metallic hazards and significant atrial or ventricular arrhythmia. Patients referred for stress testing were not recruited for this study. All participants who consented to participate completed a questionnaire for demographic information and past medical history at time of enrollment (baseline) along with a CMR exam (which included additional first-pass perfusion imaging added to the standard clinical CMR protocol). Clinical charts were reviewed to confirm cardiovascular history in all subjects. The primary outcome of interest was hospitalized heart failure after their CMR examination and was identified from electronic medical records shared by a healthcare system consisting of 6 hospitals on Long Island, NY. Vital statistics were confirmed using the National Death Index in addition to medical records.

### BNP assays

A blood sample was collected for N-terminal pro-hormone brain natriuretic peptide (NT-proBNP) before CMR for most of the study subjects. NT-proBNP was measured using an electrochemiluminescence immunoassay (Roche Diagnostics, Indianapolis, IL).

### Cardiac MRI

All participants underwent CMR on a 1.5 T scanner (Avanto, Siemens, Malvern, Pennsylvania) with an 8-element phased array surface coil. Cardiac volumes and systolic function were assessed using balanced steady-state free-precession (SSFP) cine imaging with retrospective ECG gating. Images were acquired during breath-hold in a stack of short axis planes (8 mm thickness with 2 mm gap) and 3 long axis planes (2, 3, and 4 chamber views) with the following parameters: echo time (TE) 1.3 ms, repetition time (TR) 3.1 ms, flip angle 70° and average in-plane resolution 1.3 × 1.3 mm^2^. To determine transit time, dynamic images at rest were acquired every cardiac cycle over at least 50 and up to 100 cardiac cycles in a sagittal plane where the main pulmonary artery and left atrium were well defined and additionally in a coronal view where the ascending aorta was well visualized during the infusion of 0.01 mmol/kg gadopentetate dimeglumine (Magnevist, Schering AG, Berlin, Germany) at 6 ml/s followed with a 15 ml saline flush. An ECG gated saturation recovery SSFP sequence was used with a typical field of view 500 mm, an inversion time of 90 ms, TE 0.92 ms, imaging acquisition window 160 ms per slice, slice thickness 15 mm and flip angle 50 degrees. Parallel imaging was applied with an acceleration factor of 2.

A phase-sensitive inversion recovery sequence for late gadolinium enhancement (LGE) was performed 10 to 15 min after the administration of 0.15 mmol/kg of gadopentetate dimeglumine on a stack of left ventricular (LV) diastolic short-axis slices with the following parameters: TE, 3.17 ms; flip angle, 25 degrees; voxel size, 1.9 × 1.4 mm; slice thickness, 8 mm; and field of view, 360 × 290 mm. A scout acquisition with increasing inversion time values was performed on a mid-ventricular short-axis slice to determine the inversion time that allowed for optimal nulling of normal myocardium before the LGE images were obtained.

### Image analysis

**Fig. 1:**
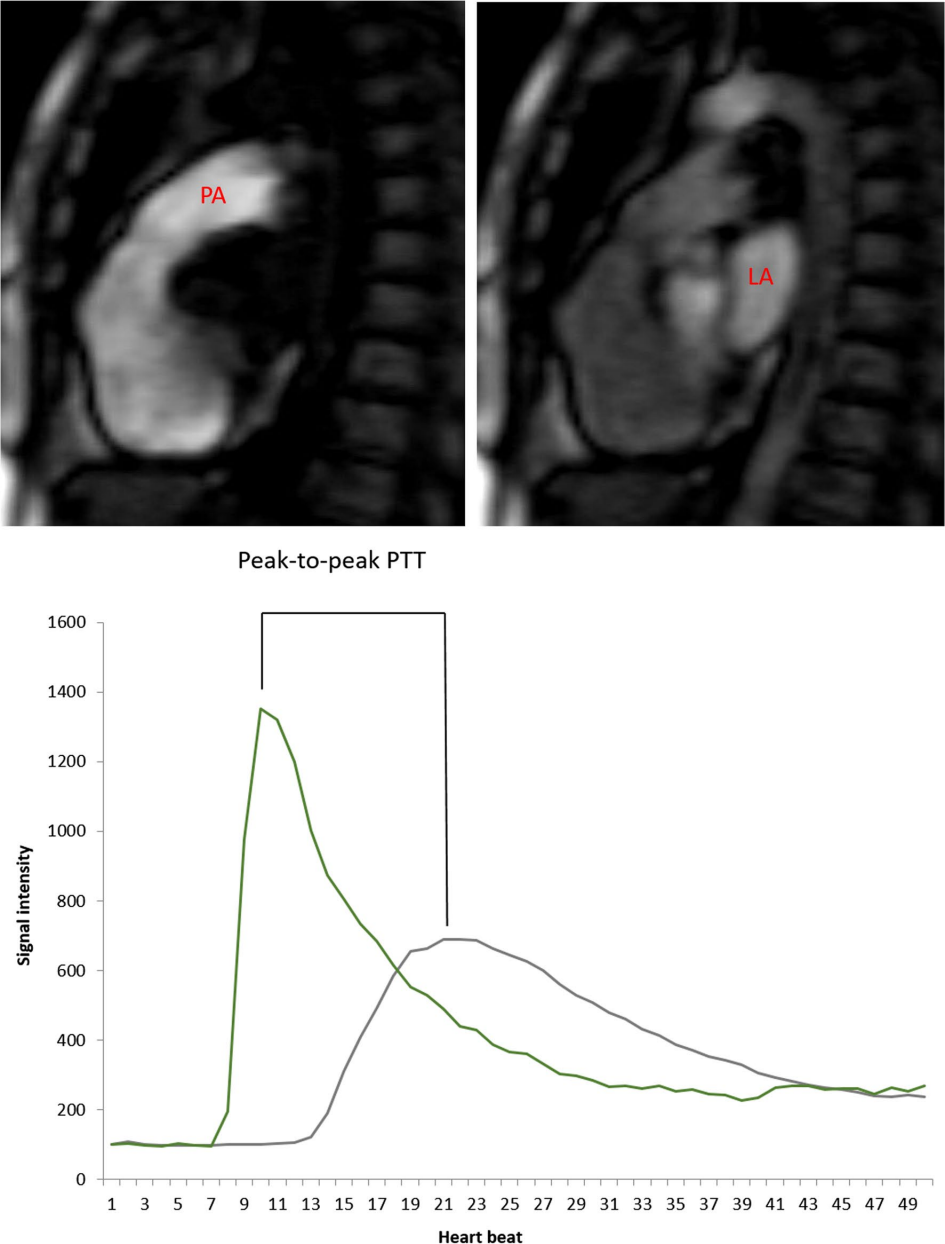
**A** Representative first pass perfusion images showing the main pulmonary artery (PA), and left atrium (LA) in standard sagittal views. **B** Example of time intensity curves obtained from main pulmonary artery (orange) and from left atrium (green) where pulmonary transit time (PTT) is calculated as the time elapsed between the two peaks

Left and right ventricular volumes, ejection fractions (EF) and myocardial mass were assessed from cine CMR images using QMASS software (Medis, Leiden, Netherlands). Left atrial volume was assessed using the biplane area-length method. LGE reading was provided by experienced imaging cardiologists following the clinical guideline [23]. CMR measurements were normalized to body surface area (BSA). PTT was calculated using the time-intensity curves generated from first-pass perfusion imaging as the peak-to-peak time difference between the main pulmonary artery and left atrium (Fig. 1) which was described with details in our previous publication [24]. In addition, PTT was also calculated using full width half maximum method. To normalize, PTT was divided by RR interval duration and can be interpreted approximately as the number of cardiac cycles required for blood to circulate between the pulmonary artery and left atrium. All the comparisons in this study were based on the normalized values unless specified otherwise.

### Statistics

Continuous variables were shown as mean ± SD for those variables with normal distribution, and median ± interquartile range (IQR) for those variables that were not normally distributed. Categorical variables were reported as frequency and percentage. PTT normalized by RR interval duration and measured during CMR at baseline was our primary exposure of interest, and time-to-first post-CMR heart failure hospitalization was our primary outcome of interest. The optimal cut point for PTT predicting heart failure hospitalization was determined by maximizing Youden’s Index applied to the receiving operator characteristic (ROC) analysis of a logistic regression model. Youden’s index defines the maximum potential effectiveness of a biomarker by combining sensitivity and specificity for all potential cut points [25]. We compared CMR measures of volume, mass, function, and LGE among subjects stratified by this optimal cut point using Student’s *t-*tests, Wilcoxon’s rank-sum test, and χ^2^ tests as appropriate.

Kaplan Meier curves were produced to examine the association between dichotomous PTT with heart failure admission over time, as well as stratified by LVEF ± 50% [26]. Hazard ratios (with 95% confidence intervals) were derived from multivariable Cox proportional hazards models examining the association of PTT (per unit or per heartbeat) increase as well as with our dichotomous cut point) with heart failure hospitalization. The models were adjusted for demographic and clinical risk factors including age, gender, body mass index (BMI), hypertension, diabetes, hyperlipidemia, smoking status, and coronary artery disease with and without additional stratification by LVEF ± 50%. A p-value < 0.05 was considered statistically significant. All analyses were performed in SAS version 9.4 (SAS Institute, Cary, North Carolina).

Several post hoc sensitivity analyses were conducted to assess the incremental benefit of improving model fit/predictability of PTT over traditional measurements of heart failure and cardiac function. Among variables consisting of left and right ventricular systolic and diastolic volumes, stroke volumes, masses, left atrial volume, fibrosis evaluated by LGE, LV global longitudinal strain, and separately BNP / NT-proBNP, we used principal component analysis to generate combinations of variables to test PTT against. We added PTT to reduced models containing these aforementioned variables in order to (1) estimate the association between PTT and hospitalized heart failure through additional adjustment for these variables and (2) assess the incremental improved model fit using the likelihood ratio test (LRT) [27] and continuous net reclassification improvement (NRI) index [28].

## Results

There were 506 consecutive subjects included in the analysis. The mean age was 63 ± 14 years and 373 (73%) were male. The mean LVEF was 49 ± 12% and there were 209 (41%) subjects with LVEF < 50%. Among diverse cardiac conditions identified by CMR the top three findings were dilated cardiomyopathy, myocardial infarction, and valvular heart disease (Table 1). After a mean follow up duration of 4.5 ± 3.0 years after their CMR exam, 70 (14%) subjects developed heart failure requiring hospitalization and among them 6 died.

**Table 1:** Baseline characteristics of participants (N = 506)

Clinical variables	Mean ± SD or N (%)
Age, years	63 ± 14
Male	373 (73)
BMI, kg/m^2^	29 ± 6
Heart rate, beats/min	69 ± 13
Diastolic blood pressure, mm Hg	75 ± 12
Systolic blood pressure, mm Hg	130 ± 19
Ever smoking	202 (39)
Diabetes mellitus	95 (19)
Hypertension	251 (50)
Myocardial infarction	71 (14)
Coronary bypass surgery	44 (9)
Coronary stents	103 (23)
History of heart failure	132 (26)
CMR findings*	
Dilated cardiomyopathy	100 (20)
Myocardial infarction	83 (16)
Hypertrophic Cardiomyopathy	49 (10)
Sarcoidosis or amyloidosis	35 (7)
Myocarditis	29 (6)
Pericardial disease	14 (3)
Valvular heart disease	109 (22)
Left ventricular hypertrophy	48 (9)
Fibrosis	86 (17)
Normal CMR	22 (4)

Date are mean ± standard deviation or n (percentage). *CMR findings total > 100% due to subjects with multiple reported findings, except for those with completely normal studies. BMI, body mass index; CMR, cardiovascular magnetic resonance

We found that RR-interval normalized PTT ≥ 8.2 was the optimal cut point for predicting hospitalized heart failure. Among those with longer PTT (≥ 8.2), left and right ventricular chamber sizes as well as left atrial size were larger, stroke volume smaller and EF lower. The prevalence of infarct scar by LGE was higher. In contrast, the prevalence of non-infarct LGE was slightly lower although it was common in both groups (Table 2).

**Table 2:** CMR findings comparing subjects with and without longer pulmonary transit time (PTT) duration

Variables	PTT < 8.2 (n = 368)	PTT ≥ 8.2 (n = 138)	p-value
LV end diastolic volume, ml/m^2^	86 ± 24	95 ± 38	0.0015
LV end systolic volume, ml/m^2^	44 ± 25	59 ± 38	< 0.0001
LV stroke volume, ml	85 ± 22	72 ± 21	< 0.0001
LV stroke volume, ml/m^2^	42 ± 10	36 ± 10	< 0.001
LV ejection fraction, %	51 ± 10	42 ± 15	< 0.0001
LV mass, g/m^2^	60 ± 16	72 ± 22	< 0.0001
RV end diastolic volume, ml/m^2^	72 ± 16	70 ± 21	0.3582
RV end systolic volume, ml/m^2^	33 ± 11	38 ± 18	0.0009
RV stroke volume, ml	77 ± 20	65 ± 19	< 0.0001
RV stroke volume, ml/m^2^	38 ± 9	32 ± 9	< 0.001
RV ejection fraction, %	54 ± 8	48 ± 11	< 0.0001
Left Atrial Volume, g/m^2^	40 ± 15	52 ± 42	< 0.001
Late gadolinium enhancement			
Infarct pattern	41 (12)	32 (23)	0.0005
Non-infarct pattern	188 (53)	61 (45)	0.0005
Both	5 (1)	7 (5)	0.0005

Date are mean ± standard deviation or n (percentage). *LV* left ventricle, *RV* right ventricle

**Fig. 2:**
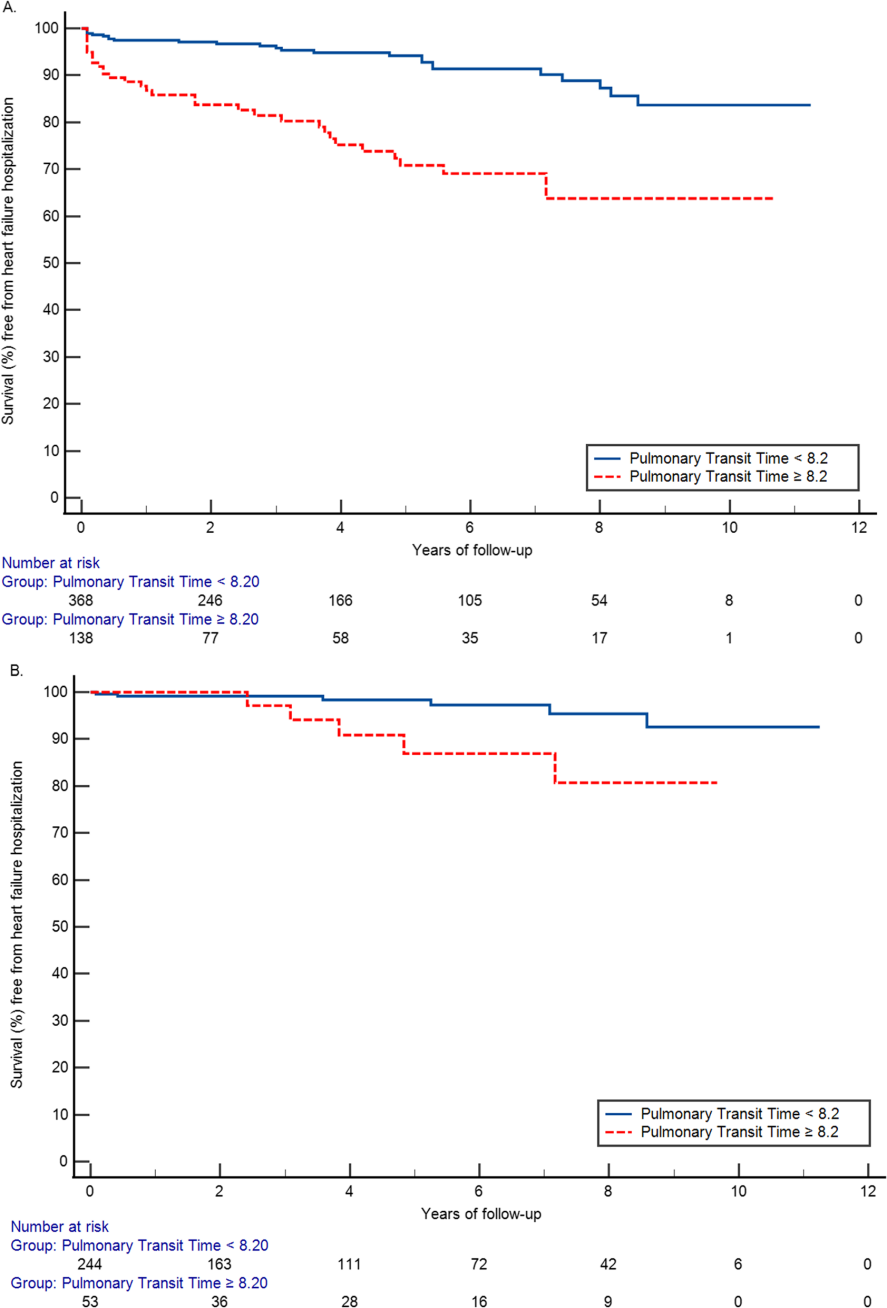

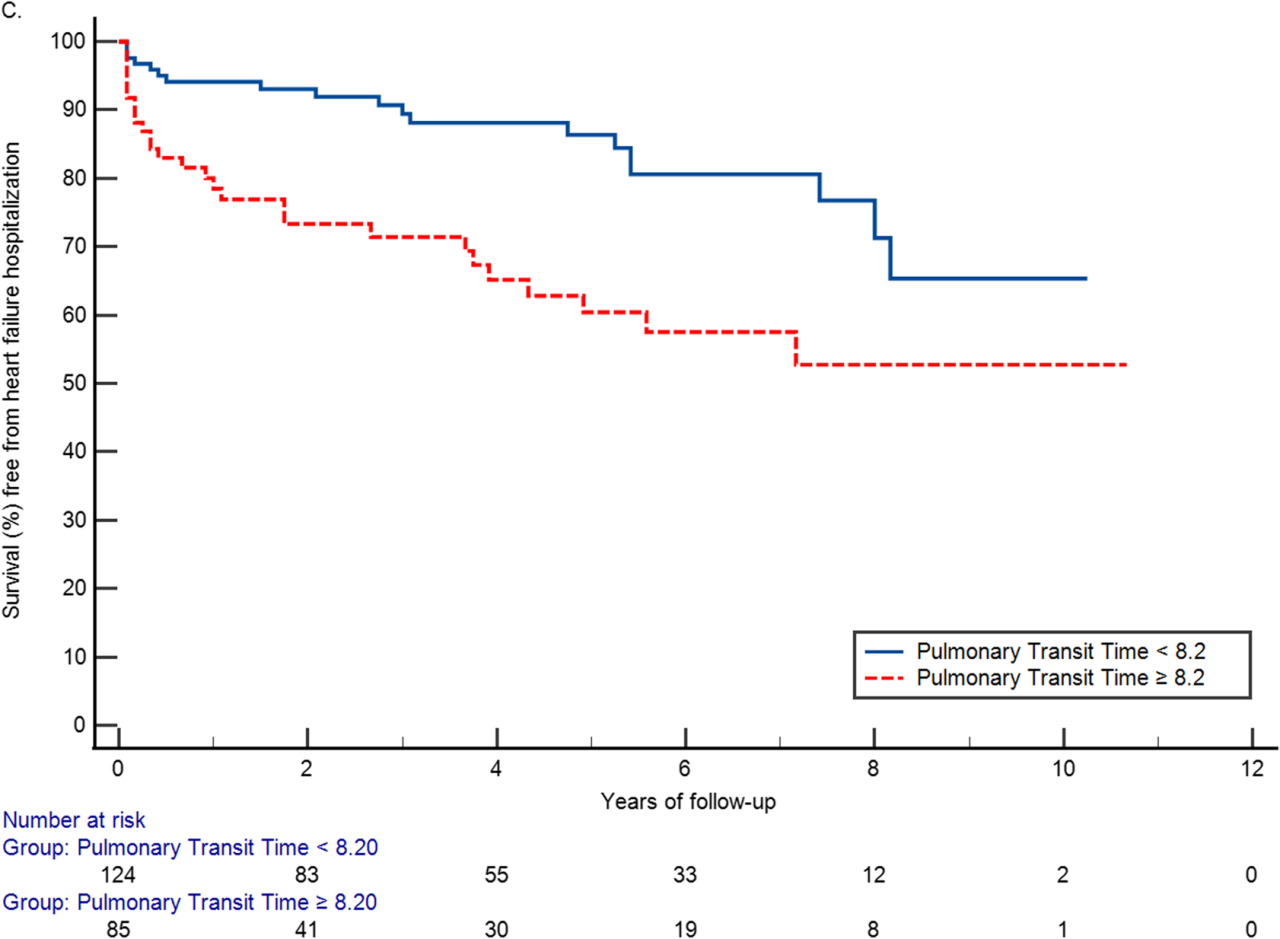
Kaplan Meier plots of incident heart failure admission over time between groups with pulmonary transit time ≥ 8.2 and < 8.2 among all patients (**A** log-rank p < 0.001), and within strata of patients with preserved left ventricular ejection fraction (**B** log-rank p = 0.0242) and patients with reduced left ventricular ejection fraction (**C** log-rank p = 0.0011)

As shown in the Kaplan Meier curves, longer PTT (≥ 8.2) was associated with reduced survival during follow up compared to those with PTT < 8.2 (Fig. 2A, log-rank p < 0.001). The difference was observed consistently in subjects with preserved and reduced LVEF (Fig. 2B and C, log-rank p = 0.0242 and p = 0.0011, respectively). In the Cox proportional hazard analysis longer PTT (≥ 8.2) was associated with a hazard ratio [95% confidence interval (CI)] of 3.90 (2.32, 6.56) when compared with a lower PTT. In the fully adjusted model including covariates of age, gender, hypertension, diabetes, smoking history, hyperlipidemia, coronary artery disease and LVEF, longer PTT remained to be an independent risk factor for heart failure with a hazard ratio of 3.69 (2.02, 6.73). Longer PTT was associated with increased hazard for both preserved and reduced LVEF yet the hazard was greater among those with preserved LVEF [HR 3.59 (1.09, 11.79)] than those with reduced LVEF [HR 2.55 (1.42, 4.56)] in the stratified analysis. The difference remained in the fully adjusted model, HR 4.18 (1.02, 17.14) vs 2.91 (1.46, 5.80), respectively.

When assessing PTT as a continuous variable in the Cox proportional hazard analysis, each 1 unit or 1 heartbeat increase in PTT was associated with a hazard ratio of 1.26 (95% CI 1.18, 1.33) in the fully adjusted model (Table 3). In the LVEF-stratified analysis, the hazard was greater among those with preserved LVEF where each unit increase of PTT was associated with 70% increase in the hazard of heart failure admission compared to 18% increase among those with reduced LVEF (Table 3) in the fully adjusted model. After additionally adjusting for LV global longitudinal strain and NT-proBNP, PTT remained an independent predictor of hospitalized heart failure (Table 3). PTT calculated using the full width half maximum method was not an independent risk factor for heart failure hospitalization (data not shown).

**Table 3:** Multivariable hazard ratios (95% confidence interval) for the pulmonary transit time normalized by RR duration (seconds) predicting heart failure admission

	All Subjects (N = 506)	LVEF < 50% (N = 209)	LVEF ≥ 50% (N = 297)
Model 1	1.22 (1.16, 1.27)	1.16 (1.08, 1.23)	1.47 (1.15, 1.90)
Model 2	1.22 (1.16, 1.29)	1.15 (1.08, 1.24)	1.42 (1.07, 1.88)
Model 3	1.26 (1.18, 1.33)	1.18 (1.09, 1.27)	1.70 (1.20, 2.41)
Model 4	1.21 (1.13, 1.29)	1.15 (1.06, 1.26)	1.87 (1.16, 3.02)
Model 5	1.19 (1.06, 1.34)	1.07 (0.94, 1.21)	1.71 (1.16, 2.52)

Model 1 = Pulmonary transit time

Model 2 = Model 1 + age + gender

Model 3 = Model 2 + BMI, hypertension, diabetes, hyperlipidemia, smoking and coronary artery disease

Model 4 = Model 3 + LV global longitudinal strain

Model 5 = Model 3 + NT-proBNP

We performed post hoc analyses to assess the incremental benefit of adding PTT to models containing known risk factors or predictors of heart failure including the estimates of cardiac function. Compared to reduced models containing baseline risk factors described above along with 1 additional measurement such as cardiac chamber size, EF, stroke volume, myocardial mass, LV global longitudinal strain, and NT-proBNP, adding PTT to these models significantly improved model fit and predictability (LRT p-values all < 0.05 and NRI demonstrating similar results). This was also true for a model with variables selected through the use of principal component analysis, whereby after adjusting for age, gender, LV end diastolic volume index, RV end systolic volume index, LA volume index, LV global longitudinal strain, and presence of LGE, PTT remained a significant risk factor for hospitalized heart failure with HR 1.15 (1.05, 1.25) per 1 unit increase and HR 1.92 (1.02, 3.61) for PTT ≥ 8.2 vs. PTT < 8.2.

## Discussion

In this prospective study, we demonstrated that longer PTT duration is an independent predictor of hospitalized heart failure in a large clinical cohort with a broad spectrum of cardiac conditions. Among those with longer PTT, there is significantly increased hazard of heart failure hospitalization with the greater hazard among those with LVEF ≥ 50%. We also demonstrated the incremental benefit of using PTT in models predicting hospitalized heart failure after considering not only traditional risk factors such as cardiac volume and function but also LV global longitudinal strain and NT-proBNP.

There is extensive literature examining the relationship of PTT with cardiomegaly, systolic dysfunction, pulmonary hypertension and heart failure due to reduced LVEF [8–17]. PTT is also correlated with B-type natriuretic peptide [29]. Recently, longer PTT duration has been observed in heart failure patients with preserved LVEF [24] as it is closely associated with altered hemodynamics such as elevated pulmonary artery wedge pressure [17, 24]. As described previously, PTT consists of 3 major components including the transit times through the right heart, the pulmonary vasculature and the left heart where the transit time through the pulmonary vasculature accounts for the largest proportion of PTT [24]. Hence, a longer PTT duration represents the collective abnormality of blood flow transit in the cardio-pulmonary circulation including the cardiac chambers and the pulmonary vasculature. It has been shown that cardiovascular risk stratification can be improved significantly when pulmonary vascular response or simply pulmonary function is incorporated into the cardiac function evaluation [30]. For example, among patients with preserved LVEF, those with increased pulmonary wedge pressure during exercise are associated with significantly increased long-term mortality risk [30]. In a large epidemiological study, the subjects with both reduced LVEF and impaired pulmonary function were associated with a far greater risk of heart failure than those with reduced LVEF or impaired pulmonary function alone [31]. The LV diastolic dysfunction algorithm recommended by the American Echocardiography Society includes pulmonary pressure as one of the four essential evaluation criteria that has effectively differentiated the risk of clinical outcome above and beyond LVEF [32–34].

There have been a number of small studies that reported outcome risk associated with longer PTT duration among patients with pulmonary hypertension [19] and those with congenital heart disease [18]. A recent publication examined a large cohort referred for chest pain evaluation using stress CMR and reported adverse clinical risk associated with longer PTT duration [20]. Similar observations were made recently among subjects with advanced heart failure [21] and with acute myocardial infarction [22]. In the present study we demonstrated in a large prospective clinical cohort with diverse common cardiac conditions that a longer PTT duration is associated with increased long-term risk of heart failure admission. Our work is an important validation of clinical relevance of assessing PTT in a general cardiac patient population thereby establishing generalizability for use with clinical cohorts. In addition, it highlights the importance of cardiopulmonary evaluation in the assessment of heart failure risk. PTT may have the potential to be complementary to routine CMR examination of cardiac structure, function and tissue characterization for heart failure risk assessment. While heart failure risk was increased independent of LVEF, the hazard ratio appears to be greater among those with preserved LVEF. We speculate that LVEF may act as an effect modifier on the relationship between PTT and heart failure risk [35]. When LVEF is reduced, LVEF presumably becomes the primary or sufficient cause of the HF hospitalizations outweighing the effect of PTT. When LVEF is preserved, PTT becomes the primary or sufficient cause of the HF hospitalizations. Our observation may have important clinical implication in the risk stratification of heart failure for patients with preserved LVEF as heart failure risk prediction for preserved EF is more complex than for those with reduced EF. In recent years, a number of powerful biomarkers have emerged in predicting heart failure risk including NT-proBNP [36, 37] and LV global longitudinal strain [38, 39]. To understand the clinical relevance of PTT, we tested the incremental value of PTT in the context of those well-established biomarkers and found PTT remains to be an important predictor of hospitalized heart failure.

Technically, PTT can be easily incorporated into routine clinical CMR examination, as the sequence of first pass perfusion is commercially available and the contrast needed is trivial. In our study we only used gadolinium contrast at 0.01 mmol/kg, a small fraction (5–6%) of the dose for late gadolinium enhancement imaging, which provides the signal intensity or image quality adequate for PTT evaluation. In addition, the time-intensity curve to calculate PTT is easily accessible using commercial software and does not require a proprietary program. Therefore, to incorporate PTT evaluation to routine clinical CMR study is feasible. We used sagittal views which are copied from the standard sagittal localizer available for all clinical CMR scans to alleviate the need for acquisition planning.

### Limitations

We acknowledge several limitations of our study. PTT is typically assessed between any representative structures of right and left circulation. The common approach is between right and left ventricle or between pulmonary artery and ascending aorta. In our study, we have chosen the main pulmonary artery and left atrium as the anatomic landmarks as they are perhaps the most appropriate chambers to mark the beginning and the end of the pulmonary circulation. Therefore, the absolute PTT value may differ from published results but the relative risk should remain unaltered. When calculating PTT, we used the peak-to-peak approach although there are several alternatives such as full width half maximum or the centroids of the time-intensity curve. Evidently, there are merits and limitations for each method. Nevertheless, we believe peak-to-peak evaluation remains the simplest and can be processed using any commercial software thereby making PTT evaluation clinically accessible. Besides technical differences, the clinical implications of PTT derived from different methods may also vary. As shown in our study, PTT calculated using full width half maximum did not seem to be associated with heart failure risk. To minimize contrast usage, we have kept the contrast dose very low with which we are able to produce adequate signal intensity for PTT analysis. However, larger dose may produce greater contrast signal and aid the post processing. Our cohort consisted of patients referred for clinical CMR with diverse cardiac conditions and levels of function thereby making the observation more generalizable comparing to studies that focus on specific cardiac conditions and types of heart failure. However, we are limited on subgroups analyses as each sub-cohort is relatively small. Lastly, this is a single center study. Future investigations with multicenter designs are warranted.

## Conclusions

To conclude, longer PTT duration can predict long-term risk of heart failure admission independent of LVEF in patients with broad spectrum of cardiac conditions. We also demonstrated the incremental benefit of using PTT in models predicting hospitalized heart failure after considering not only traditional risk factors such as cardiac volume and function but also LV global longitudinal strain and NT-proBNP. Our findings underscore the value of cardiopulmonary circulation evaluation in the heart failure risk assessment.

## Data Availability

The datasets generated and/or analyzed during the current study are not publicly available to protect patient privacy but are available from the corresponding author on reasonable request.

## Abbreviations

PTT: Pulmonary transit time
LV: Left ventricle
RV: Right ventricle
LA: Left atria
EF: Ejection fraction
CMR: Cardiac magnetic resonance
GFR: Glomerular filtration rate
HF: Heart failure
NT-proBNP: N-terminal pro-hormone brain natriuretic peptide
IQR: Interquartile range
ROC: Receiver operating characteristic
LGE: Late gadolinium enhancement
LRT: Likelihood ratio test
NRI: Net reclassification index
HR: Hazard ratio

## References

[1] Blumgart HL, Weiss S (1928). Clinical studies on the velocity of blood flow: IX. The pulmonary circulation time, the velocity of venous blood flow to the heart, and related aspects of the circulation in patients with cardiovascular disease. J Clin Invest.

[2] Freis ED, Schnaper HW, Johnson RL, Schreiner GE (1952). Hemodynamic alterations in acute myocardial infarction. I. Cardiac output, mean arterial pressure, total peripheral resistance, central and total blood volumes, venous pressure and average circulation time. J Clin Invest.

[3] Kopelman H (1951). The circulation time as a clinical test. Br Heart J.

[4] Morris LE, Blumgart HL (1957). Velocity of blood flow in health and disease. Circulation.

[5] Pearce ML, Lewis AE, Kaplan MR (1952). The factors influencing the circulation time. Circulation.

[6] Hamilton WF (1953). The Lewis A. Connor memorial lecture: the physiology of the cardiac output. Circulation.

[7] Nathanson MH, Elek SR (1947). The influence of heart size on the circulation time. Am Heart J.

[8] Shors SM, Cotts WG, Pavlovic-Surjancev B, François CJ, Gheorghiade M, Finn JP (2003). Heart failure: evaluation of cardiopulmonary transit times with time-resolved MR angiography. Radiology.

[9] Sergiacomi G, Bolacchi F, Cadioli M (2010). Combined pulmonary fibrosis and emphysema: 3D time-resolved MR angiographic evaluation of pulmonary arterial mean transit time and time to peak enhancement. Radiology.

[10] Müller HM, Tripolt MB, Rehak PH, Groell R, Rienmüller R, Tscheliessnigg KH (2000). Noninvasive measurement of pulmonary vascular resistances by assessment of cardiac output and pulmonary transit time. Invest Radiol.

[11] Skrok J, Shehata ML, Mathai S (2012). Pulmonary arterial hypertension: MR imaging-derived first-pass bolus kinetic parameters are biomarkers for pulmonary hemodynamics, cardiac function, and ventricular remodeling. Radiology.

[12] Jones RH, Sabiston DC, Bates BB, Morris JJ, Anderson PA, Goodrich JK (1972). Quantitative radionuclide angiocardiography for determination of chamber to chamber cardiac transit times. Am J Cardiol.

[13] Ohno Y, Koyama H, Nogami M (2008). Dynamic perfusion MRI: capability for evaluation of disease severity and progression of pulmonary arterial hypertension in patients with connective tissue disease. J Magn Reson Imaging.

[14] Hansch A, Heyne JP, Jung C, Wolf G, Pfeil A (2012). Quantitative first pass perfusion in cardiovascular magnetic resonance for determination of peak ventricular transit time–a technique for evaluation of heart function. Eur J Radiol.

[15] Francois CJ, Shors SM, Bonow RO, Finn JP (2003). Analysis of cardiopulmonary transit times at contrast material-enhanced MR imaging in patients with heart disease. Radiology.

[16] Saporito S, Herold IH, Houthuizen P (2016). Model-based characterization of the transpulmonary circulation by dynamic contrast-enhanced magnetic resonance imaging in heart failure and healthy volunteers. Invest Radiol.

[17] Colin GC, Pouleur AC, Gerber BL (2019). Pulmonary hypertension detection by computed tomography pulmonary transit time in heart failure with reduced ejection fraction. Eur Heart J Cardiovasc Imaging.

[18] Ait Ali L, Aquaro GD, Peritore G (2019). Cardiac magnetic resonance evaluation of pulmonary transit time and blood volume in adult congenital heart disease. J Magn Reson Imaging.

[19] Swift AJ, Telfer A, Rajaram S (2014). Dynamic contrast-enhanced magnetic resonance imaging in patients with pulmonary arterial hypertension. Pulm Circ.

[20] Seraphim A, Knott KD, Menacho K (2021). Prognostic value of pulmonary transit time and pulmonary blood volume estimation using myocardial perfusion CMR. JACC Cardiovasc Imaging.

[21] Houard L, Amzulescu MS, Colin G (2021). Prognostic value of pulmonary transit time by cardiac magnetic resonance on mortality and heart failure hospitalization in patients with advanced heart failure and reduced ejection fraction. Circ Cardiovasc Imaging.

[22] Pamminger M, Reindl M, Kranewitter C (2022). Prognostic value of pulmonary transit time by cardiac magnetic resonance imaging in ST-elevation myocardial infarction. Eur Radiol.

[23] Hundley WG, Bluemke DA, Bogaert J (2022). Society for Cardiovascular Magnetic Resonance (SCMR) guidelines for reporting cardiovascular magnetic resonance examinations. J Cardiovasc Magn Reson.

[24] Cao JJ, Li L, McLaughlin J, Passick M (2018). Prolonged central circulation transit time in patients with HFpEF and HFrEF by magnetic resonance imaging. Eur Heart J Cardiovasc Imaging.

[25] Youden WJ (1950). Index for rating diagnostic tests. Cancer.

[26] McDonagh TA, Metra M, Adamo M (2021). 2021 ESC Guidelines for the diagnosis and treatment of acute and chronic heart failure. Eur Heart J.

[27] Harrell FE (2015). Regression modeling strategies, with applications to linear models, logistic and ordinal regression, and survival analysis.

[28] Pencina MJ, D'Agostino RB, Steyerberg EW (2011). Extensions of net reclassification improvement calculations to measure usefulness of new biomarkers. Stat Med.

[29] Segeroth M, Winkel DJ, Strebel I (2023). Pulmonary transit time of cardiovascular magnetic resonance perfusion scans for quantification of cardiopulmonary haemodynamics. Eur Heart J Cardiovasc Imaging.

[30] Dorfs S, Zeh W, Hochholzer W (2014). Pulmonary capillary wedge pressure during exercise and long-term mortality in patients with suspected heart failure with preserved ejection fraction. Eur Heart J.

[31] Waheed S, Pollack S, Roth M, Reichek N, Guerci A, Cao JJ (2016). Collective impact of conventional cardiovascular risk factors and coronary calcium score on clinical outcomes with or without statin therapy: the St Francis Heart Study. Atherosclerosis.

[32] Wang L, Singh H, Mulyala RR, Weber J, Barasch E, Cao JJ (2020). The association between left ventricular diastolic dysfunction and myocardial scar and their collective impact on all-cause mortality. J Am Soc Echocardiogr.

[33] Nagueh SF, Smiseth OA, Appleton CP (2016). Recommendations for the evaluation of left ventricular diastolic function by echocardiography: an update from the American Society of Echocardiography and the European Association of Cardiovascular Imaging. Eur Heart J Cardiovasc Imaging.

[34] Nagueh SF (2018). Classification of left ventricular diastolic dysfunction and heart failure diagnosis and prognosis. J Am Soc Echocardiogr.

[35] Lash TL, Vanderweele TJ, Haneuse S, Rothman K (2021). Modern Epidemiology.

[36] van Veldhuisen DJ, Linssen GC, Jaarsma T (2013). B-type natriuretic peptide and prognosis in heart failure patients with preserved and reduced ejection fraction. J Am Coll Cardiol.

[37] Salah K, Stienen S, Pinto YM (2019). Prognosis and NT-proBNP in heart failure patients with preserved versus reduced ejection fraction. Heart.

[38] Russo C, Jin Z, Elkind MS (2014). Prevalence and prognostic value of subclinical left ventricular systolic dysfunction by global longitudinal strain in a community-based cohort. Eur J Heart Fail.

[39] Kammerlander AA, Kraiger JA, Nitsche C (2019). Global longitudinal strain by CMR feature tracking is associated with outcome in HFpEF. JACC Cardiovasc Imaging.

